# Time Trends in the Treatment and Survival of 5036 Uveal Melanoma Patients in The Netherlands over a 30-Year Period

**DOI:** 10.3390/cancers15225419

**Published:** 2023-11-15

**Authors:** Thaïs M. L. Tong, Esther Bastiaannet, Frank M. Speetjens, Christian U. Blank, Gregorius P. M. Luyten, Martine J. Jager, Marina Marinkovic, T. H. Khanh Vu, Coen R. N. Rasch, Carien L. Creutzberg, Jan-Willem M. Beenakker, Henk H. Hartgrink, Jacobus J. J. Bosch, Emine Kiliç, Nicole C. Naus, Serdar Yavuzyigitoglu, Caroline M. van Rij, Mark C. Burgmans, Ellen H. W. Kapiteijn

**Affiliations:** 1 Department of Medical Oncology, Leiden University Medical Center, Albinusdreef 2, 2333 ZA Leiden, The Netherlands; 2 Department of Radiology, Leiden University Medical Center, Albinusdreef 2, 2333 ZA Leiden, The Netherlands; 3 Department of Epidemiology, Biostatistics and Prevention, University of Zurich, Rämistrasse 71, 8006 Zürich, Switzerland; 4 Netherlands Cancer Institute, Plesmanlaan 121, 1066 CX Amsterdam, The Netherlands; 5 Department of Ophthalmology, Leiden University Medical Center, Albinusdreef 2, 2333 ZA Leiden, The Netherlands; 6 Department of Radiation Oncology, Leiden University Medical Center, Albinusdreef 2, 2333 ZA Leiden, The Netherlands; 7 Department of Surgery, Leiden University Medical Center, Albinusdreef 2, 2333 ZA Leiden, The Netherlands; 8 Centre for Human Drug Research, Zernikedreef 8, 2333 CL Leiden, The Netherlands; 9 Department of Ophthalmology, Erasmus Medical Center, Dr. Molewaterplein 40, 3015 GD Rotterdam, The Netherlands; 10 Department of Radiation Oncology, Erasmus Medical Center, Dr. Molewaterplein 40, 3015 GD Rotterdam, The Netherlands

**Keywords:** uveal melanoma, primary tumor, treatment, survival, time trends

## Abstract

**Simple Summary:**

Uveal melanoma (UM) is the most common eye tumor in adults. It is associated with dismal survival once metastasized. The treatment landscape has changed over the last years for the primary tumor, and new therapeutic options are being investigated for metastatic UM. However, it remains unclear if patients diagnosed in recent decades have a better survival compared to patients diagnosed in earlier decades. In this study, we use national data from patients diagnosed in the Netherlands between 1989 and 2019. We show that survival improvement was not related to the period of diagnosis but related to the treatment of the eye tumor with radiotherapy (for overall survival and cancer-specific survival) and female gender (for overall survival).

**Abstract:**

Background: Uveal melanoma (UM) is a rare intraocular tumor with a dismal prognosis once metastasized. This study provides a nationwide overview and time trends of patients diagnosed with primary UM in the Netherlands between 1989 and 2019. Methods: A retrospective population-based cohort study based on patients with primary UM from the database of the Netherlands Cancer Registry (NCR), linked with the national population registry Statistics Netherlands on inhabitants’ cause of death. Two time periods (1989–2004, 2005–2019) were compared with descriptive statistics. Kaplan–Meier and (multivariate) Cox proportional hazard models were used to assess changes over time for overall survival (OS) and cancer-specific survival (CSS). Results: In total, 5036 patients were analyzed with a median age of 64.0 years at the time of diagnosis. The number of patients increased over time. In the first (1989–2004) and second (2005–2019) period, 32% versus 54% of the patients received radiotherapy (*p* < 0.001). The median FU time was 13.4 years. The median OS of the first and second periods was 9.5 (95% CI 8.7–10.3) versus 11.3 years (95% CI 10.3–12.3; *p* < 0.001). The median CSS was 30.0 years (95% CI NA) in the first period and not reached in the second period (*p* = 0.008). In multivariate analysis (MVA), female gender (HR 0.85; 95% CI 0.79–0.92, *p* < 0.001) and radiotherapy treatment (HR 0.73; 95% CI 0.64–0.83, *p* < 0.001) were associated with better OS. Radiotherapy treatment (HR 0.74; 95% CI 0.61–0.90, *p* = 0.002) was also associated with better CSS. The period of diagnosis was not associated with OS or CSS. Conclusions: In this study of patients with primary UM, there was a shift to the diagnosis of smaller tumors, possibly due to stage migration. There was also an increase in eye-preserving treatments over time. OS and CSS were modestly improved in the second time period; however, the time period was not associated with OS or CSS in multivariate analyses.

## 1. Introduction

Uveal melanoma (UM) is a rare intraocular tumor that arises from melanocytes in the uveal tract. UM can develop in the choroid (90%), ciliary body (7%), or iris (3%) [1]. Despite its rarity, UM is the most common primary intraocular tumor in adults [2], with a peak incidence at 60 years of age. The highest incidences of UM are found in northern European countries, Northern USA, and Australia [3]. Aside from light skin color, risk factors include light iris color, oculodermal melanocytosis, a large amount of (atypical) skin naevi, and a germline BAP-1 mutation [2]. The incidence of UM has been stable in recent years in the US and Europe [3,4,5]. Around 30% of patients are asymptomatic at the time of diagnosis [6]. If there are symptoms, they depend on the tumor location and can present as distorted, blurred, or decreased vision or seeing flashing lights [7,8].

Once diagnosed, primary UM is treated to prevent further tumor growth and possible (worsening of) symptoms [7]. Primary treatment is with curative intent and known as ‘radical’ in case of surgical removal of the eye (enucleation) or ‘conservative’ with eye- and/or vision-sparing radiotherapeutic options [6] such as Ruthenium or Iodine plaque brachytherapy, stereotactic radiotherapy, and proton beam radiotherapy. The risk of local tumor recurrence or secondary enucleation after conservative treatment ranges from <5% up to 15%, depending on the treatment modality, size, and location of the tumor [6,9,10,11].

Following treatment for the primary tumor, five- and ten-year rates of distant metastases are 25% and 34%, respectively [12]. Up to 52% of patients will develop metastases at some time during follow-up, depending on the stage at diagnosis and the genetic constitution of the tumor [13,14,15]. Metastatic spread is primarily by hematogenous dissemination and will present in the liver in up to 90% of patients [12]. Metastasis-related mortality is high, and historically disseminated UM has been shown to be fatal within 12 months in most cases [13,16,17].

Local, liver-directed therapies may also prolong survival in patients with metastatic UM [18,19]. However, despite control of hepatic metastases after liver-directed therapy, approximately 75% of patients eventually progress with extrahepatic disease [20]. Until recently, systemic therapies have not been effective in metastatic UM. The bispecific fusion protein tebentafusp is the first drug to have shown improved 1-year overall survival (OS) in HLA-A*02:01-positive, previously untreated metastatic UM patients [21]. However, there are no standard systemic treatment options for HLA-A*02:01-negative patients, and there are only limited options available when patients show progressive disease after treatment with tebentafusp. Therefore, more treatment options are needed for patients with metastasized UM.

There are few reports with real-life data on rare cancers. This retrospective population-based cohort study provides a nationwide population-based overview and investigates time trends of patients with a primary UM diagnosis in the Netherlands between 1989 and 2019. Using data from the Dutch National Cancer Registry (NCR) and information from the Statistics Netherlands database, an overview of the treatment and survival of primary UM patients over the past 30 years is provided. Furthermore, analyses were performed comparing outcomes in the most recent diagnosis years (2005–2019) as compared to earlier decades (1989–2004), and possible factors associated with OS and cancer-specific survival (CSS) were investigated.

## 2. Materials and Methods

### 2.1. Data Retrieval

Patients with primary UM diagnosis were identified from the database of the Netherlands Cancer Registry (NCR), a nationwide population-based registry in the Netherlands with data on all cancer patients from 1989 onwards. Data are collected and managed by employees of the Netherlands Comprehensive Cancer Organization (IKNL). The NCR database contains anonymous information on newly diagnosed cancer patients, including diagnosis, tumor staging, tumor site, morphology, treatment, and survival (dead or alive) status of the patients [22]. For the purpose of this retrospective analysis, NCR data were linked with data from the national population registry Statistics Netherlands. Statistics Netherlands has information on inhabitants’ vital status and cause of death and provides microdata linkable at the individual level.

All adult patients (≥18 years) registered in the Netherlands with primary UM (stage I-IV) between 1989 and 2019 were included. Patients were not added to the NCR database in case the tumor was discovered by chance at obduction and thus was not the primary cause of death. Patients were also not included if they were living abroad at the time of primary UM diagnosis. The following data were available as coded by the NCR: gender, age at time of diagnosis, tumor location in the eye (choroid or eyeball) and tumor stage according to the American Joint Committee on Cancer (AJCC) classification (formerly, Tumor Node Metastasis (TNM) classification [23], tumor characteristics (morphology, differentiation grade), treatment (surgery, radiotherapy, other curative treatments, systemic chemotherapy, targeted therapy), follow-up time, year of death, and survival status. From the aforementioned database, patients with a confirmed primary UM diagnosis (based on clinical findings and/or histology and/or cytology) were included in this study.

Patients were excluded from our analyses if the data revealed that tumor location and morphology were unknown or uncertain or if the tumor was a non-uveal melanoma. For the deceased patients, cause of death was determined through microdata from the Statistics Netherlands database. Causes of death were registered according to the International Classification of Diseases (ICD) edition 9 for deaths occurring between 1989 and 1995 and ICD-10 for deaths from 1996 onwards. No information was available on whether/when patients developed metastatic disease or if there was local tumor recurrence. Treatment data at time of primary UM diagnosis were not available for a subgroup of patients in the database (N = 324), and subsequent treatments were also not available for this group of patients. For the scope of this study, we considered that these patients underwent active surveillance when they were included in the registry.

### 2.2. Definitions

Tumor staging was based on the TNM classification for the year in which the primary UM diagnosis was ascertained. TNM edition 4 was used from 1989 to 1998, edition 5 until 2002, edition 6 until 2009, edition 7 until 2016, and TNM 8 from 2017 onwards [23].

### 2.3. Outcome Measures

Study outcome measures were median, five-year, and ten-year OS and CSS. The OS and CSS were compared between two different time periods, 1989–2004 and 2005–2019, and adjusted for patient, uveal melanoma, and treatment characteristics in multivariate models for OS and CSS. These time periods were chosen because treatment options, especially for metastatic disease, were investigated and introduced from 2005 onwards.

### 2.4. Survival

OS and CSS were measured as the time interval from the date of the diagnosis to the date of death due to all causes, or death due to uveal melanoma or the end of follow-up, whichever came first. Patients who died due to other causes were censored. According to ICD-9 and ICD-10 classification, cancer-related deaths were defined as codes “melanoma of the skin”, “malignant neoplasms”, “neoplasms”, and “neoplasms of unspecified behavior”. Survival data were available until the end of 2021.

### 2.5. Statistical Analysis

Descriptive statistics were performed for baseline patient and tumor characteristics and were stratified according to the two subgroups based on the older and most recent years of diagnosis (1989–2004 and 2005–2019). Descriptive statistics were also used for the treatment of the primary tumor, according to the two time periods, and tested with the chi-square test. OS and CSS were analyzed for the complete cohort, as well as the two time periods (1989–2004 versus 2005–2019). The reverse Kaplan–Meier (KM) method was used to calculate median survival, including 95% confidence intervals (CI) [24]. Life tables were used to calculate 5-year and 10-year OS and CSS, including standard error (SE), and KM curves were used to depict survival curves. The log-rank test was used to compare survival curves between the periods. Univariate (UVA) and multivariate (MVA) survival analyses were performed using the Cox proportional hazards model, reporting hazard ratio (HR) and 95% CI. *p* values < 0.05 were considered statistically significant. Analyses were performed with SPSS 25.0 (SPSS Inc., Chicago, IL, USA).

## 3. Results

### 3.1. Study Population

**Figure 1:**
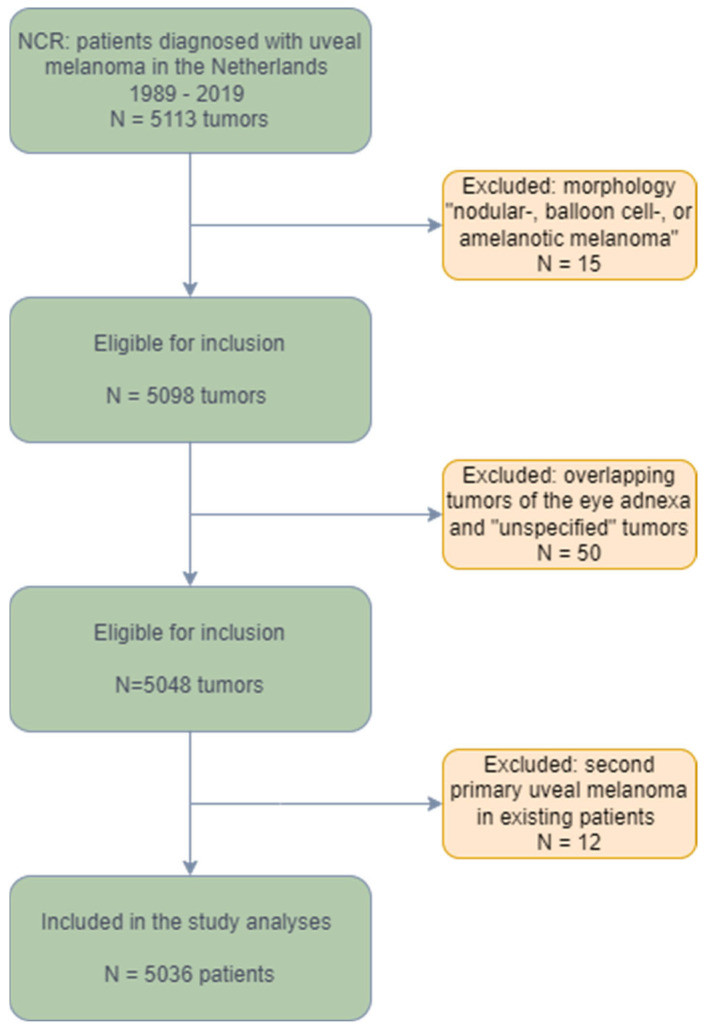
Flowchart of analyzed patients. NCR: Netherlands Cancer Registry.

Overall, 5036 patients were analyzed (see flowchart (Figure 1)). The baseline characteristics are presented for the whole cohort, as well as for the two separate time periods in Table 1. The mean age was 62.8 years, and the median age of the population was 64.0 years (range 18–96). In the total population, both gender (51% male) and affected eye (50% left eye) were equally represented. A total of 17% of the patients were diagnosed at an age younger than 50 years, 51% between the age of 50 and 70 years old, while for 32% of the population, the tumor was identified at age 70 or higher.

UM was confirmed through histological analysis in 55% of cases and by clinical and diagnostic assessment in 44% of cases. All patients had a tumor of the uvea, of which the location was the choroid (86%) or the eyeball (14%). Nineteen percent of patients were diagnosed at stage I, 47% at stage II, and 23% at stage III. Stage IV disease was detected in 3% of the population (Table 1).

**Figure 2:**
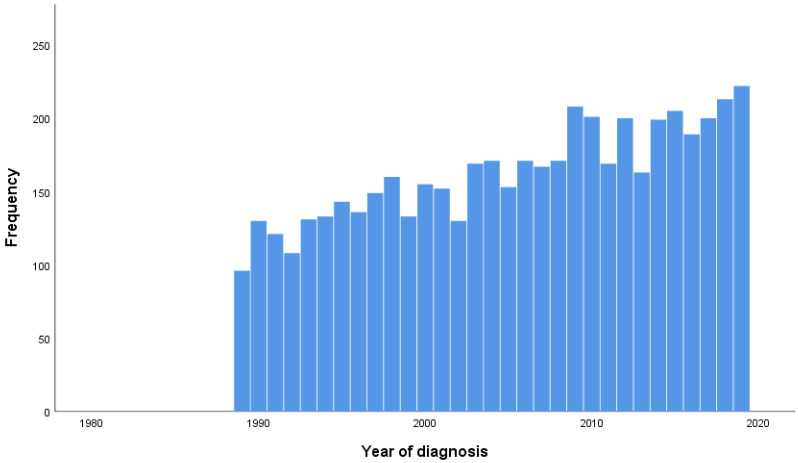
Number of new cases by year of diagnosis.

In the first time period (1989–2004), 49% of the patients were diagnosed in the age category 50–70 years and 30% at the age of 70 or higher, whereas more patients were diagnosed at higher age in the second time period, respectively, 53% and 33% in 2005–2019 (*p* < 0.001). The diagnosis was confirmed by clinical and diagnostic assessment with an increase from 32% in the first time period to 54% in the second time period. Confirmation by histological assessment was 68% in the first time period, compared to 46% in the second time period (*p* < 0.001). Regarding the disease stage, 31% of the population was diagnosed with stage II disease in the first time period, compared to 59% in the second time period. The amount of stage III and stage IV diagnoses decreased from, respectively, 33% and 5% in the first time period to 16% and 2% in the second time period (*p* < 0.001). An overview of the number of new cases by year of diagnosis between 1989 and 2019 is shown in Figure 2.

### 3.2. Treatments

The primary treatment of UM throughout the years is depicted in Table 2. Overall, surgery was performed in 51% of cases and radiotherapy in 45% of cases. Less than 1% of the population received systemic treatment with chemo- or targeted therapy, probably because of stage IV disease. For 0.3% of the population, “other curative treatments” were applied, but no specification was available on what these treatments comprised. Lastly, there was no treatment information available for 324 patients (6%). These patients were considered to have had active surveillance in follow-up. In the first time period (1989–2004), 65% of patients were treated by surgery, and 41% in the second time period (2005–2019; *p* < 0.001). Treatment with local radiotherapy modalities increased from 32% in the first period to 54% in the second time period (*p* < 0.001).

### 3.3. Overall Survival and Cancer-Specific Survival

The median follow-up (FU) time was 13.4 years (95% CI 12.8–13.9) for the whole cohort. The median FU was 22.9 years (95% CI 22.3–23.4) for the first time period and 8.2 (95% CI 7.8–8.5) for the second time period. Overall, 2692 patients (53%) died, and the cause of death was available for 2566 of these patients (95%). For 1563 of these patients (58%), the cause of death was cancer, most probably uveal melanoma.

**Figure 3:**
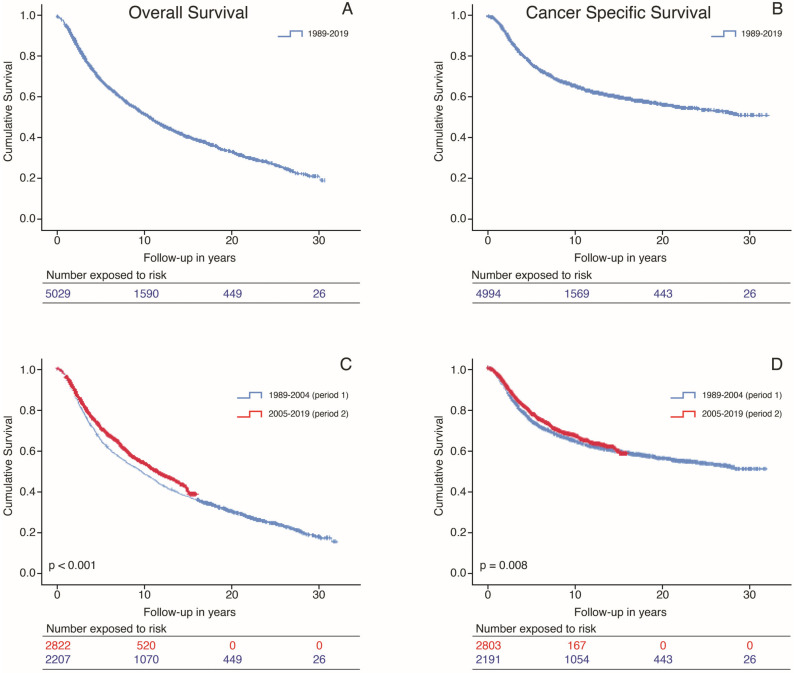
Kaplan–Meier curves representing the survival of UM patients throughout the years according to the (**A**) overall survival for the whole group, (**B**) cancer-specific survival for the whole group, and (**C**) overall survival per period of diagnosis. The curve for period 2 stops earlier due to a shorter follow-up time of that cohort. The median follow-up was 22.9 years (95% CI 22.3–23.4) for the first time period and 8.2 (95% CI 7.8–8.5) for the second time period. (**D**) Cancer-specific survival per period of diagnosis. The curve for period 2 stops earlier due to a shorter follow-up time of that cohort. The median follow-up was 22.9 years (95% CI 22.3–23.4) for the first time period and 8.2 (95% CI 7.8–8.5) for the second time period. All analyses were performed based on 5036 patients. The discrepancy between the number of analyzed patients and the number of patients exposed to risk at the start of the curves is due to the loss of patients without follow-up information.

The overall median OS was 10.5 years (95% CI 9.9–11.0) (Figure 3A). The median CSS for the whole group was 30.0 years (95% CI NA; Figure 3B).

The median OS for the cohorts (Figure 3C) diagnosed in 1989–2004 versus 2005–2019 were 9.5 years (95% CI 8.7–10.3) versus 11.3 years (95% CI 10.3–12.3; *p* < 0.001). The five- and ten-year OS for the first time period were 61% (SE 0.01) and 46% (SE 0.01), respectively. In the second time period, the five- and ten-year OS were 67% (SE 0.01) and 51% (SE 0.01) (*p* < 0.001).

The median CSS (Figure 3D) calculated for the two separate cohorts was 30.0 years (95% CI NA) for cohort 1989–2004, while the median CSS for cohort 2005–2019 has not been reached (*p* = 0.008). For the first time period, the five- and ten-year CSS were 71% (SE 0.01) and 63% (SE 0.01). This was 76% (SE 0.01) and 66% (SE 0.01) for the second time period (*p* = 0.008).

### 3.4. Variables Associated with OS and CSS in UVA

There were 26 patients who received systematic treatment with chemotherapy (n = 20) or targeted therapy (n = 6) at the time of their primary UM diagnosis, indicating that these patients had synchronous metastases. For this reason, these 26 patients were excluded from the Cox regression analyses for OS and CSS. For Cox regression analyses, clinically relevant variables were entered into the analyses. The UVA and MVA results are presented in Table 3 and Table 4.

The UVA results for the OS, including the HR and 95% CI, are shown in Table 3. For the OS, UVA showed that a higher age was significantly correlated with a worse OS (*p* < 0.001). Additionally, a higher tumor stage was correlated with a lower survival compared to stage I at diagnosis. Tumors located in the eyeball were not statistically significantly correlated with worse OS compared to tumors in the choroid (*p* = 0.69). Furthermore, surgery as a treatment for the primary tumor or “other curative treatments” were associated with poor OS (*p* < 0.001 for both variables). In contrast, radiotherapy was associated with better survival (HR 0.52; 95% CI 0.48–0.57, *p* < 0.001). Female gender (HR 0.91; 95% CI 0.84–0.98) and being diagnosed and treated in the second period (HR 0.86; 95% CI 0.79–0.93; first period as a comparison) were identified as significantly associated factors with improved OS (*p* < 0.001 for all variables).

The UVA results for the CSS are presented in Table 4. For the CSS, UVA showed higher age and higher tumor stage (*p* < 0.001 for all) at the time of diagnosis to be significantly related to a worse CSS. This was also the case for treatment with surgery and “other curative treatments” (*p* < 0.001 for both variables). Eyeball tumors were not statistically significantly correlated with worse CSS compared to tumors in the choroid (*p* = 0.74). Similar to the UVA results for OS, variables significantly related to a better CSS were primary treatment with radiotherapy (HR 0.41; 95% CI 0.48–0.57, *p* < 0.001), female gender (HR 0.88; 95% CI 0.80–0.98, *p* = 0.01), and diagnosis during the second period (HR 0.08; 95% CI 0.74–0.94, *p* = 0.003) compared to period one.

### 3.5. Variables Associated with OS and CSS in MVA

The diagnosis period was not confirmed as an independent predictor of OS in the MVA, with HR 0.94; 95% CI 0.72–1.26, *p* = 0.15. Tumor location was also not confirmed as an independent predictor of OS or CSS in MVA. Factors associated with better OS in the MVA were female gender (HR 0.85; 95% CI 0.79–0.92, *p* < 0.001) and radiotherapy as primary treatment (HR 0.73; 95% CI 0.64–0.83, *p* < 0.001). Age, higher tumor stage, surgery, and “other curative treatment” as primary treatment were associated with a worse OS (*p* < 0.001 for all; Table 3).

The period of diagnosis was not confirmed as a significant independent predictor of CSS in MVA (HR 1.09; 95% CI 0.97–1.22, *p* = 0.13). Primary treatment with radiotherapy (HR 0.74; 95% CI 0.61–0.90, *p* = 0.002) was confirmed to be related to better CSS. Female gender (HR 0.92; 95% CI 0.83–1.02, *p* = 0.12) was not significantly correlated with CSS. Furthermore, age and tumor stage at diagnosis and treatment with surgery or “other curative treatment” were significantly related to worse CSS (*p* < 0.001 for all; Table 4).

### 3.6. Adjusted Models for Period of Diagnosis in MVA

Two additional MVA models were performed to assess the factors that could statistically explain the time trends: (1) Model with period of diagnosis, adjusted for patient- and tumor characteristics (age, gender, tumor stage); (2) model with period of diagnosis, adjusted for primary tumor treatment (surgery, radiotherapy, “other curative treatment”).

When taking patient, tumor, and treatment characteristics into account in the MVA, all these characteristics show a significant correlation with OS. However, the period of diagnosis was not statistically significant. When only performing the MVA with a period of diagnosis and patient and tumor characteristics, the patient characteristics could not explain the differences in OS. However, when building the MVA model, including a period of diagnosis and adjusting for only treatment options, no statistical significance was detected for the diagnosis period, meaning that the available variables for primary treatment options could explain the difference in the OS.

The model was also built for the CSS. In this model, no statistically significant difference was detected for the diagnosis period in the first or second model, indicating that for the CSS, the patient-, tumor- and treatment-related characteristics could explain the difference in the CSS results of the two periods.

## 4. Discussion

UM is a rare cancer that has worse outcomes compared to cutaneous melanoma, especially in the advanced stage. In the past decades, treatment options have changed for the primary tumor, and new options have been introduced for the management of distant metastases. One of the aims of our retrospective study was to assess the survival of patients diagnosed with primary UM over 30 years, separated over two time cohorts that represent the introduction of other treatment modalities in more recent decades.

Firstly, our results show that there is an increase in the absolute number of new cases throughout the years. According to NCR numbers, when corrected for the population distribution with the European Standardized Rate (ESR), this corresponds with an increase of 0.71 cases per 100,000 in 1989 to 0.98 cases per 100,000 persons in 2019 [22]. Furthermore, there was an increase in diagnoses at older ages as well as an increase of stage II diagnoses in the second time period compared to the first time period. Conversely, the group diagnosed with stage III and IV disease decreased in the second time period compared to the first time period. The five-year OS was 61% in period 1 and 67% in period 2, and the ten-year OS was 46% and 51% in periods 1 and 2, respectively. The five-year CSS was 71% in period 1 and 76% in period 2, and the ten-year CSS was 63% and 66% in periods 1 and 2, respectively. Adjusted for patient, tumor, and treatment differences, the period of diagnosis was not an independent prognostic factor for OS and CSS.

### 4.1. Survival over Time

Previous studies have shown that survival rates in patients with UM have not changed much over the years [4]. Roelofsen et al. studied a large cohort consisting of more than 1000 patients treated with enucleation as primary UM treatment over five decades; there was no significant survival increase throughout the years [25]. Two meta-analyses have also identified minimal improvement in survival after diagnosis of metastatic disease, showing a median OS of 9.3 months after systemic treatments, a median OS of 14 months after liver-directed therapies, and a combined median OS of 13 months for several systemic and liver-directed therapy options [26,27]. Furthermore, analysis of OS according to published data from different studies did not show any improvement over time [27].

Our five- and ten-year survival rates are comparable to the retrospective study of Roelofsen et al. [25]. In our study, the OS in the second time period showed a modest improvement, but this was not statistically significant compared to the first time period in the MVA. Furthermore, our five- and ten-year CSS rates of 71% and 63% for period one vs. 76% and 66% for period two are within the same range found in previous studies [5,13,28]. A recent meta-analysis reported comparable combined estimates of relative survival rates of 79% at five years and 66% at 10 years [29]. A recent review indicated that the implementation of routine radiological screening at the time of primary tumor treatment in high-risk patients contributed to an improved life expectancy for patients with UM. Screening may allow earlier detection and treatment of metastases, either with liver-directed therapies and/or systemic treatments [30]. Our study results provide an indication that the primary UM diagnosis was ascertained earlier in most recent years, as there was a significant increase in the proportion of patients presenting with stage II disease in period two compared to period one, while a lower number of stage III or IV patients were seen. UM is a notoriously difficult tumor to diagnose. The symptoms that patients experience depend on the location and size of the tumor. Due to this, small tumors are often not noticed for a long period of time or are detected by chance upon performing routine eye exams. The larger proportion of lower-stage melanoma in the more recent period could indicate that there is a faster recognition of the symptoms by patients and healthcare professionals. However, the larger proportion of lower-stage melanomas could also be due to changes in the AJCC classification systems throughout the years. In the sixth edition of the TNM/AJCC classification for tumors, there have been changes in the accepted range of tumor size and height [31]. These changes led to stage migration with a decrease in stage III tumors, an increase in stage II diagnoses, and a shift from one stage to another for a selected group of patients with an impact on survival rates in both stage groups. Taking this effect into account, it is unreliable to assess the survival per stage per time period over this long registration time [32].

### 4.2. Treatment over Time

One of our study aims was also to report on treatment trends for the primary tumor in this cohort. Primary UM is treated by enucleation or with eye-conserving local radiotherapy. Since the COMS trial in 2001 determined that brachytherapy had similar survival rates when compared to enucleation, eye-conserving treatment modalities became the preferred approach of treatment for small and medium-sized tumors [14,33]. Treatment with Ruthenium or Iodine plaque brachytherapy is successful in terms of local control in up to 98% of eyes [34]. The decision on which treatment to use is based on several factors, such as tumor location, size, stage, availability of treatments, and patient preference. Large tumors are often treated with enucleation to prevent the complications related to the radiation of large tumors.

A decrease in the number of enucleations and an increase in the use of radiotherapy was observed in The Netherlands in the two described time periods (1989–2004 vs. 2005–2019), in accordance with reports in previous studies [4,7]. However, due to confounding by indication (smaller primary tumors receive radiotherapy and thus have a better prognosis), it is not possible to compare the efficacy and survival of enucleation versus radiotherapy in our study. In the past decades, therapeutic options have changed for patients who develop metastatic UM. Systemic chemotherapy does not have a place in the treatment of hepatic or extrahepatic metastases. The treatment effect is negligible, with a median OS of 10.9 months for studies concerning monotherapy or combination therapy, according to a meta-analysis [27]. Similarly, results of targeted therapies with protein kinase inhibitors such as selumetinib and AEB071 showed disappointing results [35,36,37]. Immunotherapy with immune checkpoint inhibitors (ICI) in the form of antibodies against CTLA-4, PD-1, or PD-L1 was introduced in 2010. ICI monotherapy is associated with low response rates in metastatic UM, although combination therapy with ipilimumab and nivolumab may lead to improved response rates [38,39,40,41,42,43].

Tebentafusp recognizes the glycoprotein 100 (gp100), a peptide on uveal melanoma cells that is presented by HLA-A*0201. As a result, only patients with this HLA subtype are eligible for treatment with tebentafusp [44]. Nonetheless, it is the first drug that provided increased OS in a prospective randomized trial with tebentafusp versus the investigator’s choice of treatment. Overall survival at 1 year was 73% in the tebentafusp group and 59% in the control group [21]. In our retrospective analysis, no patients were included who were treated with tebentafusp since this treatment was not yet available.

Treatment with liver-directed therapies such as isolated hepatic perfusion (IHP) or percutaneous hepatic perfusion (M-PHP) may also prolong survival in patients with hepatic metastases. Despite a response rate of 40% after IHP treatment, the treatment-related adverse events are high. Furthermore, the procedure is not repeatable. M-PHP is a repeatable, safe, and effective option in UM patients with liver metastases [20,45,46,47] and results in improved response rate, PFS, and OS compared to best alternative care [48,49]. However, a previous prospective phase II trial showed that approximately 75% of patients eventually progress with extrahepatic disease after successful initial treatment [20]. Conversely, combination therapy with ICI seems to be more effective in extrahepatic disease [41]. Based on these observations, the CHOPIN trial (NCT04283890) is currently investigating treatment with M-PHP and combined ICI, compared to M-PHP only [50].

### 4.3. Limitations

Our current retrospective cohort study provides important insights into the treatment and outcomes of patients with a primary UM diagnosis in the Netherlands over the last 30 years. However, the limitations of this study are its retrospective and anonymous nature and the fact that patient and tumor details were only registered at the moment of primary UM diagnosis. No information was available on if and when patients developed metastases. For the same reason, recurrence-free survival could also not be calculated in our study, and it was not possible to conduct sub-analyses based on treatment for metastatic spread after primary tumor detection. To determine the cause of death of patients, we relied on data from the Statistics Netherlands database on causes of death. However, while this study has its limitations, we recognize the importance of conducting epidemiological studies over a longer period of time on rare tumors such as UM to learn about changes in treatment, prognostic factors, and long-term outcomes.

## 5. Conclusions

In our retrospective cohort study over 30 years in patients with primary UM diagnosis, we show an increase in eye-preserving treatments performed over the years. Additionally, an increase in stage I/II diagnoses and higher age at the time of diagnosis are seen when comparing the time periods. Median OS and CSS have increased in period two compared to period one in the UVA, but this was not confirmed in the MVA. Different factors may have contributed to improved OS and CSS, such as earlier detection and treatment of both the primary tumor and improved therapeutic options at different stages of the disease. However, no definitive conclusions can be drawn as essential information was missing from the registry, such as the time of development and treatment of metastases. In the future, registrations should aim for the prospective collection of data, including that of treatment and outcomes after the primary diagnosis.

## Figures and Tables

**Table 1:** Baseline characteristics.

	Total N = 5036 (%)	1989–2004 N = 2209 (%)	2005–2019 N = 2827 (%)	*p*-Value
Gender				0.72
Male	2589 (51)	1142 (512)	1447 (51)	
Female	2447 (49)	1067 (48)	1380 (49)	
Age at diagnosis				<0.001
<50	857 (17)	467 (21)	390 (14)	
50–70	2580 (51)	1085 (49)	1495 (53)	
>70	1599 (32)	657 (30)	942 (33)	
Affected eye				<0.001 *
Right	2496 (49.6)	1077 (49)	1419 (50)	
Left	2519 (50)	1113 (50)	1406 (50)	
Unknown	21 (0.4)	19 (0.9)		
Diagnosis confirmation				<0.001
Clinical and diagnostic assessment	2220 (44)	698 (32)	1522 (54)	
Cytological analysis	24 (0.5)	9 (0.4)	15 (0.5)	
Histological analysis	2782 (55)	1492 (68)	1290 (46)	
Other/unknown	10 (0.2)	10 (0.5)		
Tumor location in the eye				0.006
Choroid	4305 (86)	1854 (84)	2451 (87)	
Eyeball	731 (14)	355 (16)	376 (13)	
TNM/AJCC Stage				<0.001
I	946 (19)	427 (19)	519 (18)	
II	2355 (47)	686 (31)	1669 (59)	
III	1169 (23)	727 (33)	442 (16)	
IV	163 (3)	120 (5.4)	43 (1.5)	
N/A or unknown	403 (8)	249 (11)	154 (5.4)	

AJCC = American Joint Committee on Cancer; N/A = not available; TNM = Tumor Node Metastases classification; * Significant difference due to group “unknown” in the calculation. When comparing only groups “left” and “right”, there is no statistically significant difference (*p* = 0.46).

**Table 2:** Treatments by time period.

	Total N = 5036 (%)	1989–2004 N = 2209 (%)	2005–2019 N = 2827 (%)	*p*-Value
Surgery				<0.001
Yes	2584 (51)	1437 (65)	1147 (41)	
No	2452 (49)	772 (35)	1680 (59)	
Radiotherapy				<0.001
Yes	2250 (45)	716 (32)	1534 (54)	
No	2786 (55)	1493 (68)	1293 (46)	
Systemic chemotherapy				0.73
Yes	20 (0.4)	8 (0.4)	12 (0.4)	
No	5016 (99.6)	2201 (99.6)	2815 (99.6)	
Targeted therapy				0.03
Yes	6 (0.1)		6 (0.2)	
No	5030 (99.9)	2209 (100)	2821 (99.8)	
Other curative treatment *				0.87
Yes	13 (0.3)	6 (0.3)	7 (0.2)	
No	5023 (99.7)	2203 (99.7)	2820 (99.8)	
Unknown treatment				0.67
Yes	12 (0.2)	6 (0.3)	6 (0.2)	
No	5024 (99.8)	2203 (99.7)	2821 (99.8)	
Active surveillance	324 (6.4)	147 (6.7)	177 (6.3)	

* unspecified.

**Table 3:** Cox regression analyses for OS.

Factor		Univariate Analyses			Multivariate Analyses		
		HR	95% CI	*p*-Value	HR	95% CI	*p*-Value
Age	Cont.	1.05	1.05–1.05	<0.001	1.05	1.05–1.06	<0.001
	<50	Ref.					
Age	50–70	2.32	2.03–2.65	<0.001			
	>70	5.28	4.60–6.06	<0.001			
Gender	Male	Ref.			Ref.		
	Female	0.91	0.84–0.98	0.01	0.85	0.79–0.92	<0.001
Tumor stage	I	Ref.			Ref.		
	II	1.38	1.23–1.55	<0.001	1.58	1.40–1.79	<0.001
	III	2.20	1.95–2.49	<0.001	2.07	1.82–2.34	<0.001
	IV	3.73	3.06–4.55	<0.001	2.88	2.35–3.54	<0.001
	M/Unknown	1.32	1.12–1.55	0.001	1.44	1.22–1.71	<0.001
Tumor location	Choroid(ref)/	1.02	0.92–1.14	0.69	1.02	0.91–1.14	0.789
	Eyeball						
Period	1989–2004	Ref.			Ref.		
	2005–2019	0.86	0.79–0.93	<0.001	0.94	0.72–1.26	0.15
Surgery	No (ref)/Yes	1.88	1.74–2.04	<0.001	1.39	1.22–1.60	<0.001
Radiotherapy	No (ref)/Yes	0.52	0.48–0.57	<0.001	0.73	0.64–0.83	<0.001
Other curative treatment	No (ref)/Yes	3.75	2.02–6.99	<0.001	4.29	2.30–8.01	<0.001

UVA and MVA performed in N = 5010 patients. CI = confidence interval; cont. = continuous; HR = hazard ratio; OS = overall survival; Ref. = reference.

**Table 4:** Cox regression analyses for CSS.

Factor		Univariate Analyses			Multivariate Analyses		
		HR	95% CI	*p*-Value	HR	95% CI	*p*-Value
Age	Cont.	1.02	1.02–1.03	<0.001	1.03	1.02–1.04	<0.001
	<50	Ref.					
Age	50–70	1.67	1.44–1.95	<0.001			
	>70	2.28	1.93–2.68	<0.001			
Gender	Male	Ref.			Ref.		
	Female	0.88	0.80–0.98	0.01	0.92	0.83–1.02	0.12
Tumor stage	I	Ref.			Ref.		
	II	2.20	1.83–2.64	<0.001	2.20	1.79–2.64	<0.001
	III	3.83	3.19–4.59	<0.001	3.15	2.62–3.80	<0.001
	IV	7.72	5.99–9.95	<0.001	5.53	4.27–7.17	<0.001
	M/Unknown	1.51	1.17–1.96	0.002	1.72	1.33–2.23	<0.001
Tumor location	Choroid(ref)/	1.02	0.89–1.18	0.74	1.02	0.88–1.18	0.83
	Eyeball						
Period	1989–2004	Ref.			Ref.		
	2005–2019	0.08	0.74–0.94	0.003	1.09	0.97–1.22	0.13
Surgery	No (ref)/Yes	2.74	1.74–2.04	<0.001	1.84	1.52–2.25	<0.001
Radiotherapy	No (ref)/Yes	0.41	0.48–0.57	<0.001	0.74	0.61–0.90	0.002
Other curative treatment	No (ref)/Yes	5.34	2.87–9.95	<0.001	4.46	2.38–8.33	<0.001

UVA and MVA performed in N = 5010 patients. CI = confidence interval; cont. = continuous; HR = hazard ratio; CSS = cancer-specific survival; Ref. = reference.

## Data Availability

Data were obtained from the Netherlands Cancer Registry and Statistics Netherlands. The results shown in this publication are based on calculations by Tong et al. using non-public microdata from Statistics Netherlands. The dataset is not publicly available due to the potentially identifiable nature of the data. However, data can be made available from the corresponding author upon reasonable request.
